# 简化老年综合评估在老年弥漫大B细胞淋巴瘤中应用的多中心回顾性临床研究

**DOI:** 10.3760/cma.j.cn121090-20241121-00467

**Published:** 2025-02

**Authors:** 加燕 冷, 翊鸿 蔡, 雪萍 葛, 楠平 赵, 倩倩 苏, 祝霞 贾, 军 钱, 炳宗 李, 海应 华, 旭章 卢, 华渊 朱, 建勇 李, 文瑜 施

**Affiliations:** 1 江苏大学附属人民医院血液科，镇江 212002 Department of Hematology, Affiliated People's Hospital of Jiangsu University, Zhenjiang 212002, China; 2 南京医科大学第一附属医院血液科，南京 210029 Department of Hematology, the First Affiliated Hospital of Nanjing Medical University, Nanjing 210029, China; 3 南通大学附属医院肿瘤科，南通 226001 Department of Oncology, Affiliated Hospital of Nantong University, Nantong 226001, China; 4 苏州大学附属第二医院血液科，苏州 215000 Department of Hematology, the Second Affiliated Hospital of Soochow University, Suzhou 215000, China; 5 江南大学附属医院血液科，无锡 214122 Department of Hematology, the Affiliated Hospital of Jiangnan University, Wuxi 214122, China; 6 常州市第二人民医院血液科，常州 213003 Department of Hematology, Changzhou No.2 People' s Hospital, Changzhou 213003, China

**Keywords:** 淋巴瘤，B细胞, 简化，老年综合评估, 生存分析, Lymphoma, B cell, Simplified, comprehensive geriatric assessment, Survival analysis

## Abstract

**目的:**

评价简化老年综合评估（sGA）在老年弥漫大B细胞淋巴瘤（DLBCL）患者中的预测价值。

**方法:**

回顾性分析2018年1月至2022年12月在江苏省内6家医院就诊的年龄≥60岁的初诊DLBCL患者219例，分析sGA评分与临床特征、疗效和预后的关系。

**结果:**

219例患者的中位年龄68（60～87）岁。根据sGA评分标准，将老年DLBCL患者分为适合组、不适合组和脆弱组，分别为101例（46.1％）、103例（47.0％）和15例（6.8％）。接受化疗后发生血液学不良反应最常见，三组的>2级血液学不良反应发生率相似（47.5％、41.7％和46.7％，*χ*^2^＝0.712，*P*＝0.700）。脆弱组患者的>2级胃肠道、肺不良反应及感染发生率高于适合组和不适合组，但差异无统计学意义（均*P*>0.05）。适合组、不适合组和脆弱组的缓解率分别为74.3％、46.6％和20.0％（*χ*^2^＝25.249，*P*<0.001），疾病进展率分别为5.9％、11.7％和26.7％（*χ*^2^＝6.763，*P*<0.05），2年总生存率分别为92.1％（95％ *CI* 86.6％～97.9％）、77.6％（95％ *CI* 69.5％～86.6％）、70.1％（95％ *CI* 49.4％～99.6％）（*P*<0.05），2年无进展生存率分别为76.8％（95％ *CI* 67.0％～84.8％）、69.7％（95％ *CI* 61.8％～82.0％）、65.7％（95％ *CI* 53.3％～100％），差异无统计学意义（*P*＝0.399）。

**结论:**

sGA可有效预测老年DLBCL的治疗相关不良反应、疗效、疾病进展及长期生存。

## Abstract (en)

**Objective:**

To evaluate the predictive value of simplified geriatric assessment（sGA）in elderly Chinese patients diagnosed with diffuse large B-cell lymphoma（DLBCL）.

**Methods:**

It retrospectively analyzed the relationships of sGA with the clinical characteristics, outcome, and prognosis of 219 patients aged ≥60 years who were newly diagnosed with DLBCL at six hospitals in Jiangsu province between January 2018 and December 2022.

**Results:**

The median age of 219 patients was 68 years（60–87 years）. According to the sGA system criteria, 101（46.1％）, 103（47.0％）, and 15（6.8％）elderly patients with DLBCL were categorized as fit, unfit, and frail, respectively. The most common adverse reactions after chemotherapy were hematologic, and the incidence of grade >2 hematologic adverse reactions was similar among the three groups（47.5％ *vs* 41.7％ *vs* 46.7％, respectively; *χ*^2^＝0.712, *P*＝0.700）. Compared with the fit and unfit groups, the frail group showed tendencies toward for higher proportions of grade >2 gastrointestinal, pulmonary, and infectious adverse reactions（*P*>0.05 for all）. The fit, unfit, and frail groups had respective remission rates of 74.3％, 46.6％, and 20.0％（*χ*^2^＝25.249, *P*<0.001）; disease progression rates of 5.9％, 11.7％, and 26.7％（*χ*^2^＝6.763, *P*<0.05）; 2-year overall survival rates of 92.1％（95％ *CI* 86.6％ to 97.9％）, 77.6％（95％ *CI* 69.5％ to 86.6％）, and 70.1％（95％ *CI* 49.4％ to 99.6％）（*P*<0.05）; and 2-year progression-free survival rates of 76.8％（95％ *CI* 67.0％ to 84.8％）, 69.7％（95％ *CI* 61.8％ to 82.0％）, and 65.7％（95％ *CI* 53.3％ to 100％）（*P*＝0.399）.

**Conclusion:**

sGA can effectively predict treatment adverse effects and efficacy, disease progression, and long-term survival in elderly DLBCL.

弥漫大B细胞淋巴瘤（DLBCL）是最常见的淋巴瘤亚型。西方国家中位发病年龄为67岁，约29％的患者≥75岁[1]。DLBCL的一线标准方案为R-CHOP方案，5年生存率可达64.7％。与年轻患者相比，老年DLBCL具有独特的生物学特征，且合并症多、预后较差，65岁以上患者5年总生存（OS）率仅为55.1％[2]。与年轻患者相比，老年患者面临着更多与年龄相关的疾病，而这不仅减少他们接受治疗的机会，同时也增加了治疗相关不良反应的发生风险。因老年患者在临床试验中的代表性不足，其治疗决策仅根据医师的经验决定。年龄通常是降低标准化疗强度的主要原因[3]，然而部分70～80岁的患者可以从标准化疗中获益[4]。多方位评估老年患者的状态，并根据评估结果制定不同层次的治疗方案，是改善老年DLBCL患者预后的有效途径。老年综合评估（CGA）是OS的独立预测因素，可以有效地用于老年DLBCL患者的治疗决策。近年来，意大利淋巴瘤基金会在原版CGA[5]体系上提出改进，创建新的简化老年综合评估（sGA）体系，用于预测老年DLBCL的预后，并首次将其与国际预后指数（IPI）及HGB结合构建了新的老年DLBCL的预后模型，即老年预后指数（EPI）[6]，引起广泛关注。本研究利用sGA对江苏省内6家医院的老年DLBCL患者的预后进行评估。

## 病例与方法

1. 病例资料：回顾性收集2018年1月至2022年12月江苏省淋巴瘤协作组的6家医院共219例年龄≥60岁新诊断的DLBCL患者的连续临床资料。诊断标准参照世界卫生组织（WHO）2022版淋巴造血系统肿瘤分类标准[7]，所有入选病例均经2名病理学专家共同诊断。纳入标准：①初诊患者；②具有明确的病理诊断。排除标准：①临床资料不完整；②原发中枢神经系统淋巴瘤；③转化的DLBCL。

2. sGA体系：如表1所示，sGA体系包括年龄、日常生活能力（ADL）[8]、工具性日常生活能力（IADL）[9]以及老年累积疾病评定量表（CIRS-G）[10]。按照评分体系将患者分为适合组、不适合组及脆弱组。

**表1:** 简化老年综合评估分组标准

标准	适合组	不适合组		脆弱组
ADL	≥5分	<5分	6分	<6分
IADL	且≥6分	或<6分	且8分	或<8分
CIRS-G	且无3～4级合并症（且2级合并症≤8个）	或3～4级合并症≥1个（或2级合并症>8个）	且无3～4级合并症，2级合并症<5个	或3～4级合并症≥1分，2级合并症≥5个
年龄	<80岁	<80岁	≥80岁	≥80岁

**注** ADL：日常生活能力评定量表；IADL：工具性日常生活能力评定量表；CIRS-G：老年累积疾病评定量表

3. 治疗方案：标准治疗方案为R-CHOP21［利妥昔单抗375 mg/m^2^第0天；环磷酰胺750 mg/m^2^，第1天；多柔比星50 mg/m^2^或表柔比星70 mg/m^2^或脂质体多柔比星30 mg/m^2^，第1天；长春新碱1.4 mg/m^2^（不超过2 mg/d）或长春地辛4 mg，第1天；泼尼松100 mg/d，第1～5天；3周为1个周期］。根据整个疗程（6个周期）中每例患者实际的蒽环类给药剂量与理论的给药剂量的比值，对治疗方案进行分类：①全剂量治疗：以蒽环类药物为基础的化疗组合（由CHOP或CHOP样方案与利妥昔单抗组成），蒽环类实际给药剂量≥理论给药剂量的70％；②减低剂量治疗：蒽环类实际给药剂量<理论给药剂量的70％；③姑息治疗：不含蒽环类药物的治疗方案，包括单纯放射治疗、不含蒽环类药物［COP（环磷酰胺+长春新碱+泼尼松）、低剂量COP］的低剂量化疗、利妥昔单抗单药、糖皮质激素单药、口服单药化疗。标准蒽环类药物剂量强度定义为6个周期的多柔比星50 mg/m^2^或表柔比星70 mg/m^2^或脂质体多柔比星30 mg/m^2^，第1天，3周为1个周期，没有任何延迟或减量。由于死亡或疾病进展（PD）导致蒽环类治疗少于6个周期的病例，也被认为是符合蒽环类标准剂量强度[11]。

4. 定义与评估：患者的体能状态通过美国东部肿瘤协作组（ECOG）积分评估。日常活动能力通过ADL和IADL评估。合并症通过CIRS-G和查尔森合并症指数（CCI）[12]评估。治疗相关不良反应采用WHO标准评估。>2级血液学不良反应的评估标准为出现WBC<2.0×10^9^/L或中性粒细胞计数<1.0×10^9^/L或HGB<80 g/L或PLT<50×10^9^/L。总反应率（ORR）定义为完全缓解（CR）率+不确定的完全缓解（CRu）率+部分缓解（PR）率。营养风险评估采用老年营养风险指数（GNRI）。GNRI的计算公式为14.89×白蛋白（g/dl）+41.7×体重/理想体重[13]。

5. 随访：以查阅门诊和住院电子病历及电话联系相结合的方式进行。截止日期为2023年7月31日。中位随访时间为32（4～67）个月。无进展生存（PFS）期定义为从诊断到出现复发、进展或死亡的时间，对于未出现复发、进展或死亡的患者，观察截至末次随访时间。总生存（OS）期定义为从确诊到因为任何原因死亡的时间或末次随访时间。

6. 统计学处理：统计分析采用SPSS 27.0软件、Graphpad prism 10.1软件和R 4.4.2软件。计数资料使用例（％）描述。计量资料使用*M*（范围）描述。连续变量的组间比较采用Wilcoxon秩和检验（两组）或Kruskal-Wallis检验（两组以上）。组别间的分类变量比较采用非参数或Fisher精确检验。单因素生存分析采用Kaplan-Meier统计法，采用Log-rank分析比较精算生存曲线。不受干预因素影响的完全随机缺失值，采用均值进行填补，分类变量的缺失处理，将缺失值单独列为一组进行计算。使用受试者工作特征（ROC）曲线和曲线下面积（AUC）分别评估sGA、CGA和EPI的敏感性和特异性，使用对数秩检验进行组间比较。*P*≤0.05为差异有统计学意义。

## 结果

1. sGA分组与临床特征：收集江苏省内6家医院的具有完整的CGA数据的219例患者临床资料。根据sGA体系标准，适合组、不适合组和脆弱组分别有101例（46.1％）、103例（47.0％）和15例（6.8％）。年龄<80岁的201例患者：适合组患者共101例，均为无3～4级合并症的患者，47例（46.5％）为ADL和IADL均无失能，47例（46.5％）仅在IADL中有1～2项失能，3例（3.0％）仅在ADL中有1项失能，4例患者同时存在ADL的1项失能和IADL的1～2项失能；不适合组患者共100例，均为无>8个2级合并症的患者，12例（12.0％）ADL和IADL均无失能，但存在至少1个3～4级合并症。年龄≥80岁的患者共18例，其中3例为不适合组患者，其ADL和IADL评分均无失能，无3～4级合并症。其余15例在一种或两种评估量表中存在失能或有3～4级合并症，归为脆弱组。

所有患者的临床特征如表2所示，男124例（56.6％）、女95例（43.4％），中位年龄68（60～87）岁。适合组的年龄、ECOG评分、IPI评分、结外受累≥1处的比例、骨髓受累的比例、HGB较不适合组和脆弱组差异均具有统计学意义（均*P*<0.05）。

**表2:** sGA体系分组的老年弥漫大B细胞淋巴瘤患者的临床资料［例（％）］

临床特征	总体（219例）	适合组（101例）	不适合组（103例）	脆弱组（15例）	*χ*^2^值	*P*值
年龄［岁，*M*（范围）］	68（60～87）	71（60～79）	70（60～82）	81（80～87）	48.042	<0.001
男性	124（56.6）	57（56.4）	62（60.2）	5（33.3）	3.849	0.146
non GCB亚型^a^	112（51.1）	50（51.5）	56（54.9）	6（40.4）	4.091	0.394
B症状	56（25.6）	21（20.8）	33（32.0）	2（13.3）	4.656	0.097
ECOG评分≥2分^b^	76（34.7）	16（16.0）	49（48.0）	11（73.3）	33.017	<0.001
Ann Arbor分期Ⅲ～Ⅳ期	149（68.0）	62（61.4）	75（72.8）	12（80）	4.123	0.127
结外受累≥1处^c^	74（33.8）	24（24.2）	44（42.7）	6（40.0）	7.918	0.019
骨髓受累^d^	43（19.6）	12（12.0）	27（26.5）	4（28.6）	7.336	0.026
LDH（>1 ULN）	104（47.5）	45（44.6）	51（49.5）	8（53.3）	0.720	0.698
IPI评分^e^					19.718	<0.001
低危（0～1分）	42（19.2）	28（27.7）	14（13.6）	0（0）		
低中危（2分）	51（23.3）	29（28.7）	20（19.4）	2（14.3）		
高中危（3分）	41（18.87）	19（18.8）	18（17.5）	4（28.6）		
高危（4～5分）	84（38.4）	25（24.8）	51（49.5）	8（57.1）		
HGB≥120 g/L	114（52.1）	64（63.4）	44（42.7）	6（40.0）	9.605	0.008
GNRI^f^					3.893	0.143
无危险（>98分）	113（51.6）	59（58.6）	49（48.0）	6（40.0）		
轻度危险（92～98分）	42（19.2）	19（19.2）	19（18.6）	4（26.7）		
中度危险（82～92分）	46（21.0）	17（17.2）	26（25.5）	3（20.0）		
重度危险（<82分）	15（6.8）	5（5.1）	8（7.8）	2（13.3）		
β_2_微球蛋白≥2.8 mg/L	93（42.5）	38（37.6）	45（43.7）	10（66.7）	4.607	0.100
CCI评分					11.121	0.004
0～1分	170（77.6）	87（86.1）	70（68.0）	13（86.7）		
2分	28（12.8）	10（9.9）	17（16.5）	1（6.7）		
≥3分	21（9.6）	4（4.0）	16（15.5）	1（6.7）		
发生>2级不良反应						
血液	98（44.7）	48（47.5）	43（41.7）	7（46.7）	0.712	0.700
胃肠道	15（6.8）	8（7.9）	5（4.9）	2（13.3）	1.813	0.404
肺	12（5.5）	6（5.9）	4（3.9）	2（13.3）	2.334	0.311
感染	18（8.2）	8（7.9）	8（7.8）	2（13.3）	0.560	0.756

**注** sGA：简化老年综合评估；non GCB：非生发中心B细胞来源；ECOG：美国东部肿瘤协作组；IPI：国际预后指数；GNRI：老年营养风险指数；CCI：查尔森合并症指数；ULN：正常值上限；^a^207例可供分析；^b^217例可供分析；^c^217例可供分析；^d^216例可供分析；^e^217例可供分析；^f^216例可供分析

2. sGA分组与不良反应：在219例患者中，接受化疗后发生>2级的血液学不良反应最常见（98例，44.7％），其次为感染（18例，8.2％）和胃肠道反应（15例，6.8％）。适合组、不适合组和脆弱组的>2级血液学不良反应的发生率相似（*P*＝0.700）。脆弱组的>2级胃肠道、肺部不良反应及感染发生率稍高于适合组和不适合组，但差异均无统计学意义（均*P*>0.05）（表2）。

3. sGA分组与治疗反应：所有患者的CR+CRu率、PR率和ORR分别为57.5％、27.4％、84.9％。适合组、不适合组和脆弱组的CR+CRu率分别为74.3％、46.6％、20.0％，差异具有统计学意义（*χ*^2^＝25.249，*P*<0.001）；两两比较，适合组的CR+CRu率高于不适合组（*χ*^2^＝16.292，*P*<0.001）和脆弱组（*χ*^2^＝17.455，*P*<0.001），不适合组的CR+CRu率亦高于脆弱组，但差异无统计学意义（*χ*^2^＝3.776，*P*＝0.052）。三组的PD率分别为5.9％、11.7％、26.7％，差异具有统计学意义（*χ*^2^＝6.763，*P*<0.05）；两两比较，适合组的PD率低于不适合组（*χ*^2^＝2.067，*P*＝0.151）和脆弱组（*χ*^2^＝7.122，*P*<0.05），不适合组的PD率低于脆弱组，但差异无统计学意义（*χ*^2^＝2.519，*P*＝0.112）。

4. 老年综合评估和EPI对生存的预测能力：依据sGA体系，适合组、不适合组、脆弱组的2年OS率分别为92.1％（95％ *CI* 86.6％～97.9％）、77.6％（95％ *CI* 69.5％～86.6％）、70.1％（95％ *CI* 49.4％～99.6％）（*P*＝0.022）（图1A）。两两比较，适合组的2年OS率高于不适合组（*P*＝0.009），而不适合组与脆弱组差异无统计学意义（*P*>0.05）。三组的2年PFS率分别为76.8％（95％ *CI* 67.0％～84.8％）、69.7％（95％ *CI* 61.8％～82.0％）、65.7％（95％ *CI* 53.3％～100％），差异无统计学意义（*P*＝0.399）（图1B）。

**图1:**
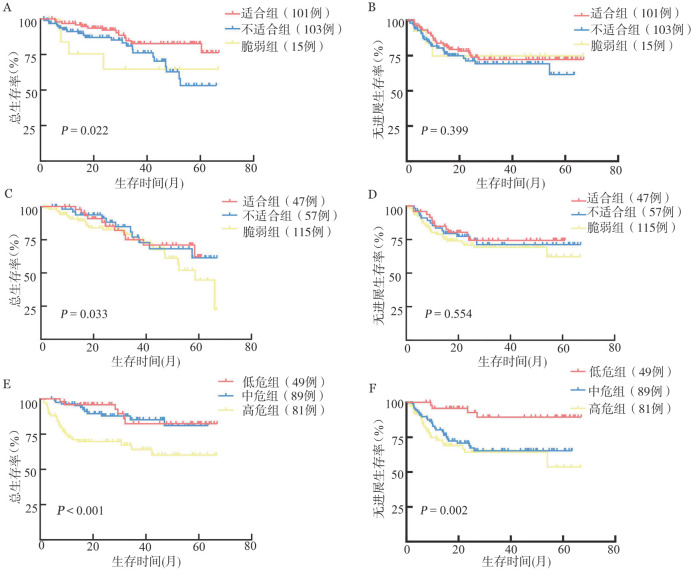
基于老年综合评估和老年预后指数（EPI）对老年弥漫大B细胞淋巴瘤患者的生存分析 **A、B** 基于简化老年综合评估体系分组的老年弥漫大B细胞淋巴瘤患者的总生存和无进展生存曲线；**C、D** 基于老年综合评估体系分组的老年弥漫大B细胞淋巴瘤患者的总生存和无进展生存曲线；**E、F** 基于EPI分组的老年弥漫大B细胞淋巴瘤患者的总生存和无进展生存曲线

同时，我们还依据了原版CGA的分组体系，分析了适合组（47例）、不适合组（57例）以及脆弱组（115例）的生存。三组2年OS率分别为92.1％（95％ *CI* 83.8％～100％）、90.4％（95％ *CI* 82.8％～98.8％）、77.0％（95％ *CI* 69.3％～85.6％）（*P*＝0.033）（图1C）。2年PFS率分别为74.4％（95％ *CI* 62.2％～89.0％），77.4％（95％ *CI* 66.8％～89.0％），71.0％（95％ *CI* 62.1％～81.1％）（*P*＝0.554）（图1D）。适合组的2年OS率高于脆弱组（*P*＝0.023），但适合组与不适合组间、不适合组与脆弱组间的2年OS率差异均无统计学意义（均*P*>0.05），由此可见原版CGA未能明显区分三组间的OS。

依据EPI评分，低危组（49例），中危组（89例），高危组（81例）的2年OS率分别为98.0％（95％ *CI* 94.1％～100.0％）、87.8％（95％ *CI* 80.6％～95.7％）、69.7％（95％ *CI* 60.0％～81.22％）（*P*<0.001）（图1E）。2年PFS率分别为92.8％（95％ *CI* 80.6％～100％）、69.0％（95％ *CI* 59.5％～80.1％）、64.3％（95％ *CI* 53.0％～78.0％）（*P*＝0.002）（图1F）。高危组的2年OS率低于中危组（*P*<0.001）和低危组（*P*＝0.002），但低危组与中危组差异无统计学意义（*P*＝0.491）；低危组的2年PFS率高于中危组（*P*<0.001）和高危组（*P*＝0.002），但中危组与高危组差异无统计学意义（*P*＝0.443）。

ROC曲线显示，sGA（*P*＝0.001）、CGA（*P*＝0.004）和EPI（*P*<0.001）对于老年DLBCL患者的2年OS率预测均具有统计学意义。sGA的AUC为0.657（95％ *CI* 0.563～0.751，*P*＝0.001），CGA的AUC为0.638（95％ *CI* 0.543～0.733，*P*＝0.001），而EPI的AUC为0.715（95％*CI* 0.632～0.799；*P*<0.001）。EPI在2年OS率预测方面优于CGA（*P*＝0.050）。sGA和EPI在OS预测方面差异无统计学意义（*P*＝0.111），sGA和CGA在OS预测方面差异也无统计学意义（*P*＝0.377）（图2A）。另外，ROC曲线显示，EPI对于老年DLBCL患者PFS预测具有统计学意义（*P*<0.001），EPI的AUC为0.642（95％ *CI* 0.558～0.727，*P*<0.001）。而sGA（*P*＝0.391）和CGA（*P*＝0.348）均无法预测老年DLBCL患者的PFS（图2B）。

**图2:**
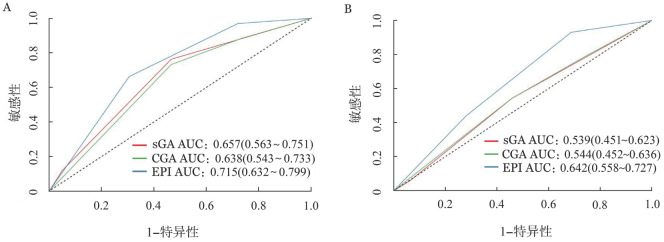
预测老年弥漫大B细胞淋巴瘤患者的总生存（A）和无进展生存（B）的受试者工作特征（ROC）曲线 **注** sGA：简化老年综合评估；CGA：老年综合评估；EPI：老年预后指数；AUC：曲线下面积

对常见影响DLBCL生存时间的因素进行单因素生存分析显示，ECOG评分、Ann Arbor分期Ⅲ～Ⅳ期、LDH≥1 ULN、IPI评分、GNRI、β_2_微球蛋白、ADL、IADL、3～4级合并症个数、2级合并症个数、治疗强度均为患者生存时间的影响因素（均*P*<0.05）。

5. 基于sGA体系的分层治疗可行性分析：根据临床医师的经验选择治疗方案，适合组中的101例患者中有62例接受全剂量治疗、35例接受减低剂量治疗、4例患者接受姑息治疗。接受姑息治疗的4例中，1例接受奥妥珠单抗+利妥昔单抗+泽布替尼治疗、1例接受利妥昔单抗+GemOx（吉西他滨+奥沙利铂）治疗、1例接受利妥昔单抗+来那度胺+GemOx治疗，还有1例为原发胃DLBCL，手术切除病灶后因患者拒绝标准方案化疗，予R-COP方案治疗，随访55个月至截止日期持续获得良好的生活质量。3种治疗方案的CR率分别为79.0％、68.6％、50.0％（*χ*^2^＝3.314，*P*＝0.507）；PD率分别为3.2％、8.6％、25.0％（*χ*^2^＝3.852，*P*＝0.146）。全剂量治疗组的CR率高于减低剂量治疗组和姑息治疗组，PD率低于减低剂量治疗组和姑息治疗组，差异无统计学意义。全剂量治疗组与减低剂量治疗组的3年OS率分别为90.2％（95％ *CI* 81.1％～100％）、68.1％（95％ *CI* 51.3％～90.4％）（*P*＝0.016），3年PFS率分别为77.2％（95％ *CI* 66.9％～89.1％）、64.2％（95％ *CI* 48.9％～84.2％）（*P*＝0.301），由于姑息治疗组样本量较小，故未纳入分析中。

不适合组（103例）中48例接受全剂量治疗、33例接受减低剂量治疗、22例接受姑息治疗。三种治疗方案的CR率分别为58.3％、48.5％、18.2％（*χ*^2^＝9.842，*P*＝0.007）。全剂量治疗组（*χ*^2^＝5.238，*P*＝0.022）和减低剂量治疗组（*χ*^2^＝9.800，*P*＝0.002）的CR率均高于姑息治疗组，但全剂量治疗组与减低剂量治疗组的CR率差异无统计学意义（*χ*^2^＝0.764，*P*＝0.382）。三组的PD率分别为2.1％、12.1％、31.8％（*χ*^2^＝12.969，*P*＝0.002）。姑息治疗组的PD率高于全剂量治疗组（*χ*^2^＝13.177，*P*<0.001）。减低剂量治疗组的PD率高于全剂量治疗组（*χ*^2^＝3.420，*P*＝0.065），姑息治疗组PD率高于减低剂量组（*χ*^2^＝3.201，*P*＝0.074），但差异均无统计学意义。三组的3年OS率分别为76.1％（95％ *CI* 63.7％～90.9％）、83.5％（95％ *CI* 71.2％～97.9％）、40.6％（95％ *CI* 16.8％～98.3％）（*P*＝0.057）。姑息治疗组的3年OS率低于减低剂量治疗组（*P*＝0.046）和全剂量治疗组（*P*＝0.035），但减低剂量治疗组与全剂量治疗组差异无统计学意义（*P*＝0.520）。三组的3年PFS率分别为71.4％（95％ *CI* 58.6％～87.0％）、73.9％（95％ *CI* 58.6％～93.3％）、52.8％（95％ *CI* 30.3％～92.2％）（*P*＝0.311）。因此，在不适合组中，相较于接受姑息治疗的患者，接受全剂量治疗或减低剂量治疗的患者可获得更高的CR率和更低的PD率，且在OS和PFS上均有获益。

脆弱组（15例）无接受全剂量治疗的患者，9例接受减低剂量治疗、6例接受了姑息治疗，CR率分别为33.3％和0（*χ*^2^＝2.500，*P*＝0.114），PD率分别为22.2％和33.3％（*χ*^2^＝0.227，*P*＝0.634）。脆弱组患者接受减低剂量治疗和姑息治疗的3年OS率分别为76.2％（95％ *CI* 52.1％～100％）和60.0％（95％ *CI* 29.3％～100％），3年PFS率分别为100％（95％ *CI* 100％～100％）和64.8％（95％ *CI* 39.3％～100％）。减低剂量组3年OS和PFS率略高于姑息治疗组，但差异均无统计学意义（均*P*>0.05）。

## 讨论

本研究参照了意大利淋巴瘤基金会的一项前瞻性多中心临床研究[6]使用的sGA体系，根据ADL、IADL、CIRS-G对患者进行分组，报道了中国东部地区老年DLBCL的sGA分布概况。结果显示，在219例年龄≥60岁的DLBCL患者中，适合组、不适合组和脆弱组分别占46.1％、47.0％、6.8％。与意大利的sGA分布相比，不适合组比例较高、脆弱组比例较低，这可能与我们的研究对象中>80岁的患者比例较低有关。同时我们也将这些患者进行了CGA分组，根据CGA体系，适合组、不适合组、脆弱组分别为47例（21.5％）、57例（26.0％）、115例（52.5％）。因sGA的评分标准较CGA放宽，所以CGA中有70％的病例被重新分配，其中100例CGA认定为脆弱的患者在sGA中被划归为不适合，另有54例CGA认定不适合的患者被划归为sGA中的适合。

本研究表明，sGA分组对于老年DLBCL的疗效、PD和预后有着有效的预测能力。与以往研究相似[6]，本研究结果显示，适合组的缓解率高于不适合组与脆弱组，PD率低于不适合组和脆弱组，2年OS率高于不适合组和脆弱组。Tucci等[5]的研究采用CGA体系也得到了类似的结论，适合组的2年OS率显著高于不适合组+脆弱组。Bai等[14]采用Tucci的CGA体系将老年DLBCL患者分为适合组、不适合+脆弱组。结果提示适合组的CR率及3年OS率均高于不适合+脆弱组。2021年意大利淋巴瘤基金会在原版CGA基础上优化了分组方法，将年龄≥65岁的老年DLBCL按照新版sGA体系分组，结果显示适合组、不适合组、脆弱组3年OS率分别为75％、58％、43％。与原版CGA相比，sGA体系能更好地区分出不适合组和脆弱组之间的差异[6]。本研究中不适合组的OS率和PFS率虽高于脆弱组，但差异无统计学意义，这可能与治疗方案有关。脆弱组中，9例使用了含蒽环类的减量方案，余6例使用了不含蒽环类的治疗方案，包括1例未化疗、1例接受R+GemOx方案治疗、2例接受利妥昔单抗+来那度胺+COP方案治疗、1例接受利妥昔单抗+奥布替尼+COP方案治疗、1例接受利妥昔单抗+来那度胺+COP+替雷利珠单抗方案治疗。这些免疫调节剂、小分子靶向药物及低毒的化疗方案可能延长了患者的生存时间。上海交通大学医学院附属瑞金医院开展的一项Ⅱ期临床研究[15]中纳入了30例经CGA评估为不适合/脆弱的初治DLBCL患者，予以无化疗方案（利妥昔单抗+来那度胺+伊布替尼），中位随访27.6个月，2年OS率和PFS率分别为100％和88.9％。江苏省人民医院的单中心前瞻性研究[16]显示，60～70岁且ECOG评分>2分但无严重合并症或>70岁的DLBCL患者接受R-GemOx方案治疗，3年OS率和PFS率分别为65.0％和49.0％。这些临床研究也证实了含吉西他滨的方案以及免疫调节剂、布鲁顿酪氨酸激酶抑制剂等新药的使用在老年无法耐受标准治疗的患者中取得了较好的疗效，另一方面提示该评估体系可能不完全适用于中国的老年DLBCL患者。除了sGA体系外，其他参与CGA的指标也值得关注，例如评价躯体功能的客观且简便易操作的指标步速、握力，评价营养状态的客观指标GNRI以及社会经济支持等可纳入CGA体系中。Liu等[17]报道步速可以独立预测老年血液肿瘤患者的生存和住院率，改善患者评估、预后和个体化护理。Lee等[18]报道GNRI不仅是OS的预测指标，而且与CCI联用时，可显著提高对预后的预测准确率，具有对老年DLBCL进行预后分层的能力。本研究单因素生存分析结果也提示GNRI可影响患者预后，即GNRI<82分组（12例）2年OS率低于GNRI≥82分（196例）（38.9％对86.3％，*P*<0.001）。

在sGA体系指导治疗的可行性分析中，本研究结果显示适合组中接受全剂量治疗的患者3年OS率高于减低剂量治疗组，CR率高于减低剂量治疗组和姑息治疗组，PD率低于减低剂量治疗组和姑息治疗组，说明适合组患者接受全剂量治疗更能获益。姑息治疗组4例患者3年OS率为100％，分析原因仍然与我们的治疗方案相关，并且该组例数较少，结果的参考价值有限。不适合组患者的减低剂量治疗组和全剂量治疗组3年OS率高于姑息治疗组，说明不适合组患者接受全剂量或减低剂量治疗比姑息治疗更能获益。脆弱组患者接受减低剂量治疗与姑息治疗的3年OS率及PFS率差异均无统计学意义。总体而言，根据sGA分组制定化疗方案具有一定的可行性，但仍需要开展更多的前瞻性临床研究以探索依据CGA选择治疗方案的临床策略。

综上，我们在国内多家医疗中心开展的大规模回顾性研究中发现，sGA可以预测老年DLBCL的疗效、PD及预后，单因素分析提示多种因素可影响患者预后。期待将来开展更大样本量多中心前瞻性临床研究，以完善适合中国老年DLBCL的CGA体系，并据此制定老年DLBCL的治疗方案。
